# Isothermal crystallization kinetics of (Cu_60_Zr_25_Ti_15_)_99.3_Nb_0.7_ bulk metallic glass

**DOI:** 10.1038/s41598-020-67390-y

**Published:** 2020-06-29

**Authors:** Soumen Mandal, Dong-Eun Lee, Taejoon Park

**Affiliations:** 1 Intelligent Construction Automation Center, Kyungpook National University, 80, Daehak-ro, Buk-gu, Daegu, 41566 Republic of Korea; 2 School of Architecture and Civil Engineering, Kyungpook National University, 80, Daehak-ro, Buk-gu, Daegu, 41566 Republic of Korea; 3 Department of Robotics Engineering, Hanyang University, 55 Hanyangdaehak-ro, Ansan, Gyeonggi-do 15588 Republic of Korea

**Keywords:** Engineering, Materials science

## Abstract

This paper reports the crystallization kinetics of (Cu_60_Zr_25_Ti_15_)_99.3_Nb_0.7_ bulk metallic glass under isothermal conditions. Differential scanning calorimetry (DSC) has been employed for isothermal annealing at ten different temperatures prior to the onset of crystallization (*T*_*o*_) temperature. X-ray diffraction and transmission electron microscopy have been used to confirm the amorphous structure of the as cast sample. Crystallized volume fractions (*x*) are calculated from the exothermic peaks of DSC scans. Crystallized volume fractions (*x*) against time show sigmoidal type of curves as well as the curves become steeper at higher annealing temperatures. Continuous heating transformation diagram has been simulated to understand the stability of the bulk metallic glass. Crystallization kinetics parameters are calculated using Arrhenius and Johnson–Mehl–Avrami equations. Activation energy (*E*_a_) and Avrami exponential factor (*n*) have exhibited strong correlation with crystallized volume fraction (*x*). The average activation energy for isothermal crystallization is found to be 330 ± 30 kJ/mol by Arrhenius equation. Nucleation activation energy (*E*_*nucleation*_) is found to be higher than that of growth activation energy (*E*_*growth*_). The Avrami exponential factor (*n*) indicates about the diffusion controlled mechanism of the nucleation and three-dimensional growth.

## Introduction

The lack of long-range order in the atomic assembly makes the bulk metallic glasses (BMGs) unique with the combination of properties viz. high strength, low elastic modulus, large elasticity, high fracture toughness and exceptional corrosion resistance^1–9^. Therefore, BMGs are considered to be promising structural materials in different engineering fields^10^. The estimation of the thermal properties of any material is very crucial before its application as engineering materials. BMGs are in metastable state and transform to the crystalline state on heating beyond the super-cooled liquid region^11^. The structural and functional properties significantly change with crystallization of the BMGs^12,13^. For instance, the magnetic properties have been reported to improve in Fe-based metallic glasses by the formation of nanocrystals inside the amorphous matrix^14,15^. Colored metallic glass has been reported by Na et al. for Au-based composites consisting of a metallic glass matrix and finely dispersed gold microdendrites (MGMCs)^16^. The improvement of the mechanical properties in BMG is also reported by creating second phase inside the amorphous matrix^17,18^. Yan et al. (2015) reported about the stress-induced nano-crystallization at the shear plane due to the localized heating in Zr_60_Al_15_Ni_25_ BMG^19^. Metastable B2 reinforced BMG composite of Zr_48_Cu_47.5_Al_4_Nb_0.5_ alloy was reported with strong strain-hardening capability and large elastic strain limit by Wu et al.^20^. Formation of nanocrystals inside the glass matrix in Fe-based BMGs due to the addition of Ni has been reported to increase plasticity in good extent^21^. The second phase inside the amorphous matrix restricts the rapid propagation of the shear bands and induces the shear bands to multiply or branching, that ultimately results in higher plastic strain^22^. The nano-crystals, crystalline particles are considered to be the second phase inside the glass matrix^22^.

Isothermal crystallization kinetics can give an idea about the time and temperature for isothermal heating of BMGs to create a second phase of the desired size which can result into a ductile BMG. Therefore, the understanding about the crystallization kinetics of BMGs is very important. The crystallization kinetics in amorphous materials is sensitive to the parameters such as; activation energy, nucleation and growth mechanism, crystalline temperature and time, nature of the crystalline phases being formed^6^. The phase transformation process can be divided into two individual stages; (i) formation of nucleation, and (ii) nucleation growth and the crystal phase stability^23^. The nucleation stage implicates the formation of embryos whereas, at the growth stage, the size of the nuclei is increased to stable crystalline phases.

Last few decades, Cu-based bulk metallic glasses (BMGs) have been of great research interest worldwide due to its high glass forming ability, good mechanical properties, high corrosion resistance and reasonable materials cost compare to other BMG systems^10,24–26^. Excellent mechanical properties^27,28^ along with a high glass forming ability^29,30^ make Cu- based glassy alloys important candidates for practical applications in structural areas such as “microengineering”, for example the fabrication of micrometric parts^31,32^. However, the thermoplastic forming of Cu-based BMGs can lead to the crystallizations. Therefore, also to avoid the crystallization during the high-temperature processing viz. thermoplastic forming (TPF), the detailed understanding of crystallization kinetics of (Cu_60_Zr_25_Ti_15_)_99.3_Nb_0.7_ BMG has been the subject of this study. There are many available reports on the glass forming ability, thermal, mechanical and corrosion properties of Cu-BMGs^33–36^. No reported study was found on the isothermal crystallization kinetics on the Nb added Cu-Zr-Ti BMG. Earlier study showed the enhancement of plasticity by 16% for the Nb added Cu_60_Zr_25_Ti_15_ BMG^37^, thus, it is utmost required to understand about the isothermal kinetics of the present alloy for its application. In the present paper, isothermal crystallization kinetics of (Cu_60_Zr_25_Ti_15_)_99.3_Nb_0.7_ BMG has been investigated by DSC at different temperatures prior to the onset of the crystallization temperature. The different kinetic parameters are calculated in order to explain details of the nucleation and growth behaviors during the crystallization processes. A continuous heating transformation diagram has been plotted from the isothermal crystallization data to understand the stability of the (Cu_60_Zr_25_Ti_15_)_99.3_Nb_0.7_ BMG.

## Experimental procedure

The master alloy with nominal composition of (Cu_60_Zr_25_Ti_15_)_99.3_Nb_0.7_ (at.%) was prepared by arc melting of the mixture of high purity elements (99.9% Cu, 99.5% Zr, 99.95% Ti, and 99.8% Nb, by mass) under Ti-gettered high purity argon atmosphere. The alloy ingot was remelted for five times to homogenise the composition. The alloy was remelted and suction cast into water-cooled copper mold to cast the BMG plate of 1 mm thickness and 8 mm breadth. The structure of the as- cast and annealed samples were analysed by the X-ray diffractometer [Bruker D8 Discover] with Cu K_α_ radiation. The structure of the as-cast sample was reconfirmed by transmission electron microscopy (TEM) [JEOL- 2,200 FS]. Differential scanning calorimetry (DSC) [Perkin-Elmer Diamond DSC] analysis was performed in isochronal conditions under the continuous flow (30 ml/min) of purified Ar. Temperature and the enthalpy calibrations prior to the DSC experiments were done using Indium and Zinc. For minimizing the effect of the structural relaxation, the samples were annealed to a set temperature at a heating rate of 40 K/min, and further, kept at the selected temperatures for a certain period until the completion of the crystallization. The volume of the crystalline phase (*x*) at time *t* was calculated based on the fractional area of the exothermic event. Johnson–Mehl–Avrami (JMA) approach was used to calculate the crystallization activation energies under isothermal conditions and pre-exponential factors.

## Result and discussion

### X-ray diffraction

**Figure 1:**
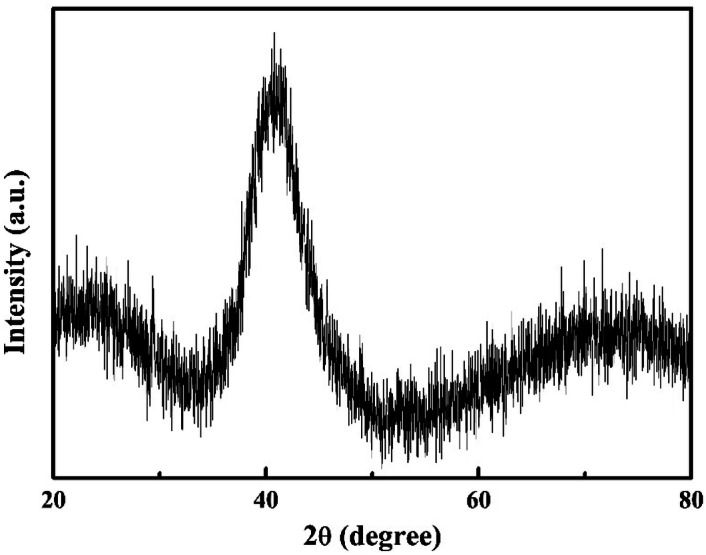
X-ray diffractogram of as cast (Cu_60_Zr_25_Ti_15_)_99.3_Nb_0.7_ BMG plate.

The X-ray diffraction pattern of the as quenched (Cu_60_Zr_25_Ti_15_)_99.3_Ni_0.7_ BMG plate (Fig. 1) shows a broad diffraction halo, indicating the amorphous state of the samples.

### Transmission electron microscopy

**Figure 2:**
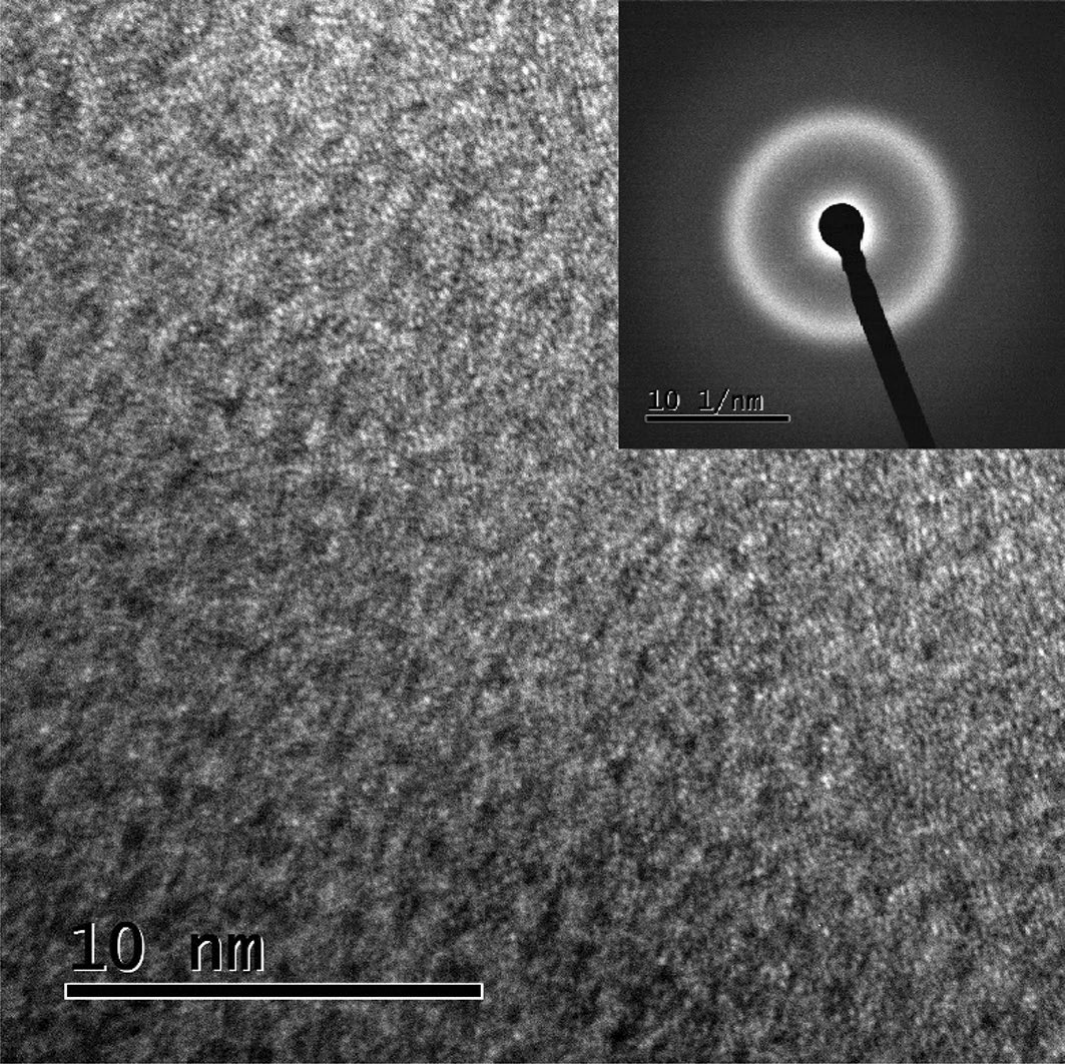
TEM micrograph and SAED pattern (inset) of as cast (Cu_60_Zr_25_Ti_15_)_99.3_Nb_0.7_ sample.

TEM microstructure and the corresponding selected area electron diffraction pattern (SAEDP) of as-cast (Cu_60_Zr_25_Ti_15_)_99.3_Ni_0.7_, presented in Fig. 2, exhibits typical salt pepper contrast. This microstructure and the diffused rings of the SAEDP further confirm the amorphous nature of the BMG plate.

### Isothermal crystallization

**Figure 3:**
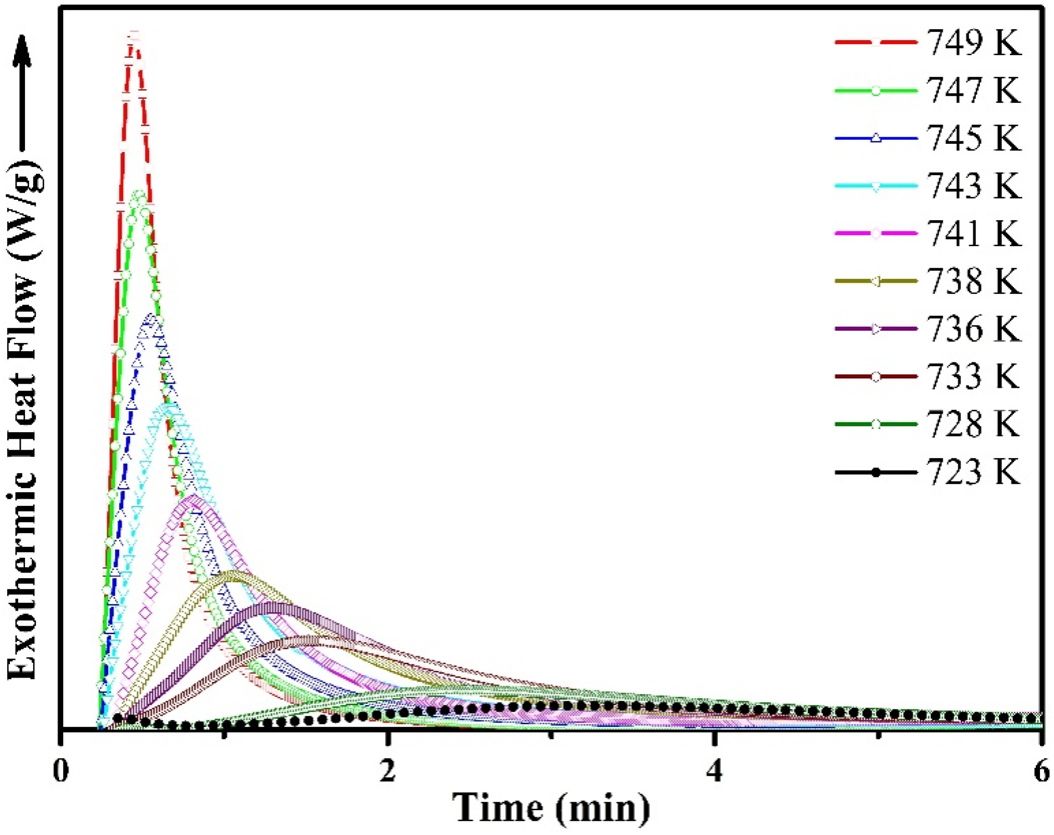
Isothermal DSC curves of (Cu_60_Zr_25_Ti_15_)_99.3_Nb_0.7_ bulk metallic glass at different annealing temperature.

The isothermal crystallization DSC curves of kinetics of (Cu_60_Zr_25_Ti_15_)_99.3_Ni_0.7_ BMG at different annealing temperatures are shown in Fig. 3. The DSC curves exhibit single exothermic peaks afterwards a particular incubation period, τ. It can be seen from the DSC curves that, with the increase in the annealing temperature, the incubation time decreases. This phenomenon can be explained as the higher mobility of the atoms inside the alloy at higher annealing temperature yields a critical fluctuation in concentration to enable the long range ordering of atoms for crystallization in larger scale^24,38,39^. Simultaneously, the width of the exothermic peak increases significantly with the increasing incubation period which demonstrates about more sluggish crystallization process. The similar observation for Cu-based bulk metallic glasses have been reported earlier^24,40^.

**Figure 4:**
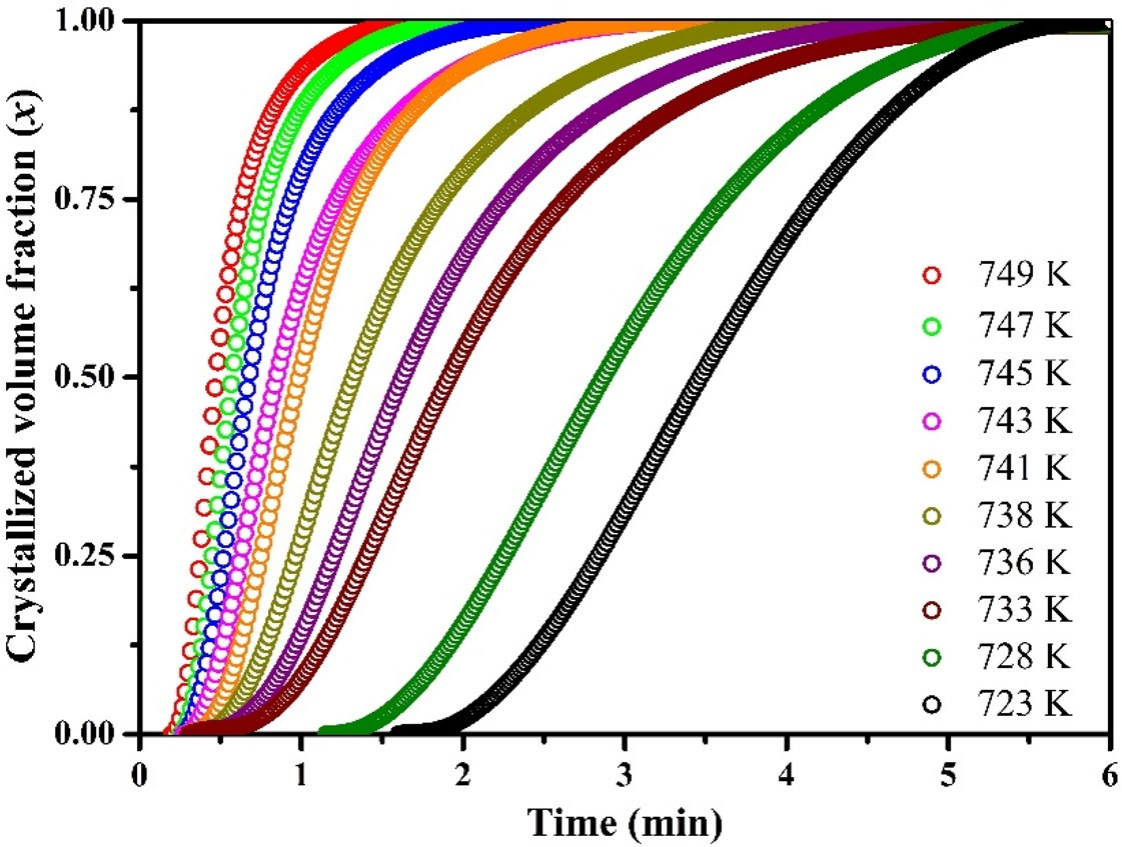
Plots of relationship between the crystallized volume fraction *x* and annealing time *t* at different annealing temperatures.

It is well known that the crystallization volume fraction is directly proportional to the fractional area of the peak area of exothermic heat flow. So, it is easy to calculate the accurate crystallized volume fraction by measuring the partial area of the exothermic heat flow curves. From the isothermal DSC curves presented in Fig. 3, the progression of the crystalline volume fraction versus the annealing time has been calculated and presented in Fig. 4. All the curves show a typical sigmoid progression as well as the curves are steeper at the higher annealing temperature. Eventually, the samples transform from a metastable state to stable state^39,41^.

#### Activation energy

**Figure 5:**
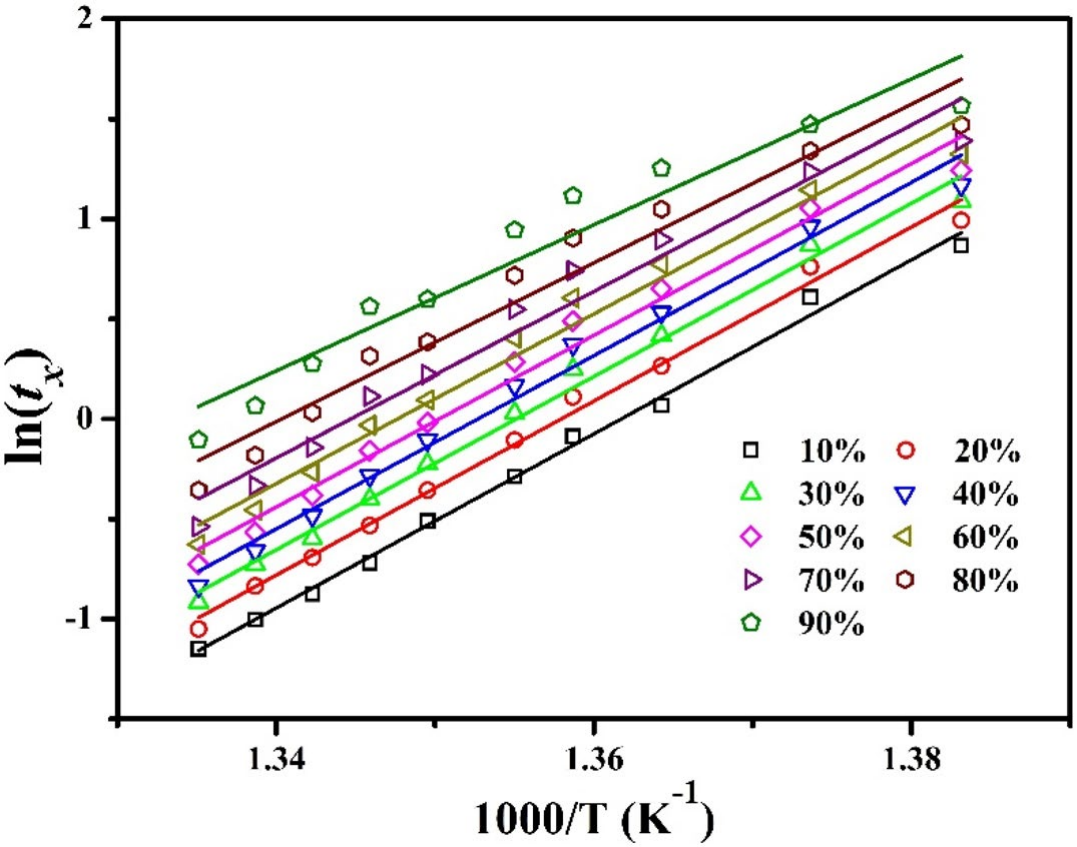
Plots for activation energy determination in isothermal conditions.

The understanding of the activation energy is very crucial to understand the crystallization process. The local activation energy *E*_a_ for the isothermal crystallization process can be determined using the Arrhenius equation^24,39,42^:

1 $$t_{x} = t_{0} \exp \left( {\frac{{E_{{\text{a}}} }}{RT}} \right)$$

where *t*_*x*_ is the annealing time required for the crystallization volume fraction to *x*, *t*_*0*_ is a constant, and *R* is universal gas constant (*R* = 8.314 J mol^−1^ K^−1^). *T* represents the annealing temperature. The plots of ln(*t*_*x*_) versus 1000/T at different crystallized volume fractions (at 10% to 90%) are shown in Fig. 5. Using Arrhenius equation, the *E*_a_ can be obtained by fitting a straight line to the experimental values of different volume fractions.

The relation between the local activation energy (*E*_a_) and crystallized volume fraction (*x*) has been presented in graphical form in Fig. 5. The figure clearly exhibits that the crystallization process starts at x < 10% as well as the local activation energy (*E*_a_) finally decreases with increasing the crystallized volume fraction. The *E*_a_ value is almost constant at ± 360 kJ/mol upto 50% crystallized volume fraction and then finally decreases to 303 kJ/mol. This behavior demonstrates that the crystallization process becomes easier with annealing time or with the progressive crystallized volume fractions (*x*). This may be conferred as the energy requisite at the initial stage for the nucleation (*E*_*nucleation*_), continuously decreases with the forward of crystallization process and energy is consumed only for growth (*E*_*growth*_) process^24,43^.

**Figure 6:**
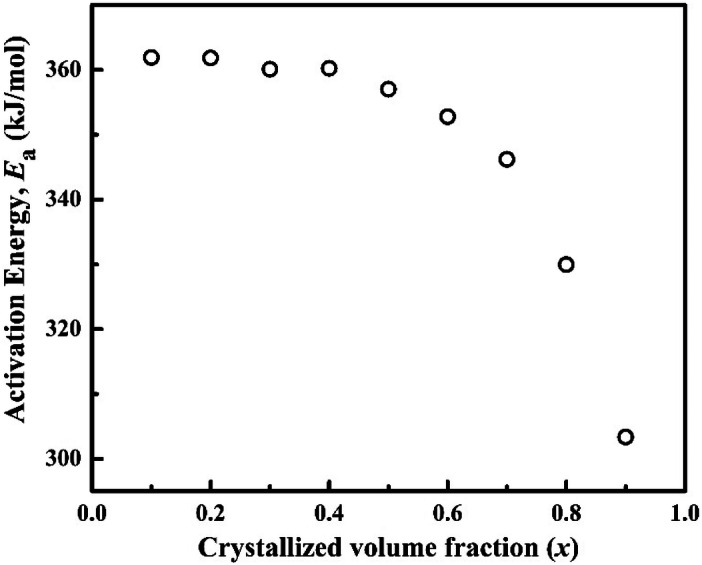
Crystallized volume fraction, *x* versus apparent activation energy, *E*_a_.

It is considered that the higher activation energy (*E*_a_) indicates the higher energy required for the nucleation during quenching. So, higher *E*_a_ demonstrates the difficulty for nucleation i.e.; the higher glass forming ability (GFA) of the alloy systems. The activation energies reported for Mg_61_Cu_28_Gd_11_, (Mg_61_Cu_28_Gd_11_)_98_Cd_2_, and (Mg_61_Cu_28_Gd_11_)_99.5_Sb_0.5_ alloys are 88. 132, and 112 kJ/mol, respectively^44,45^. For Cu_55_Zr_45_ metallic glass the *E*_a_ reported to vary from 181.1 to 187.8 kJ/mol in isothermal crystallization process^46^. The *E*_a_ values in isothermal crystallization mode for other BMG systems viz. Ca_65_Mg_15_Zn_20_ (123.5 kJ/mol)^47^, Ce_70_Ga_6_Cu_24_ (91–148 kJ/mol)^48^, Ti_16.7_Zr_16.7_Hf_16.7_Cu_16.7_Ni_16.7_Be_16.7_ (259.9 kJ/mol)^49^, Cu_47.5_Zr_47.5_Al_5_ (285 kJ/mol)^50^^,^ Zr_60_Cu_25_Fe_5_Al_10_ (325 kJ/mol)^51^ are lesser than the *E*_a_ value obtained in the current study (Fig. 6). So, the GFA of the (Cu_60_Zr_25_Ti_15_)_99.3_Ni_0.7_ alloy pretends more compared to those reported alloy systems.

#### Crystallization mechanism

The isothermal crystallization process in BMGs can be described by the Johnson–Mehl–Avrami (JMA) model by plotting the isothermal annealing time and crystallized volume fraction (*x*). The JMA equation can be expressed as^43,47,52,53^:

2 $$x(t) = 1 - \exp [ - K(t - \tau )^{n} ]$$

where, *x* represents the crystallized volume fraction, t indicates about the time required for the crystallization of volume faction *x*, *τ* is the incubation period before the start of nucleation, and *K* is a rate constant as function of temperature. Furthermore, *n* is the Avrami exponent and it is related to the nucleation and growth mechanism. The values of *n* and *K* can be determined by taking double logarithm of the JMA equation. The modified JMA equation is expressed as follows^6,24,39,47^:

3 $$\ln \{ - \ln [1 - x(t)]\} = n\ln k + n\ln (t - \tau )$$

**Figure 7:**
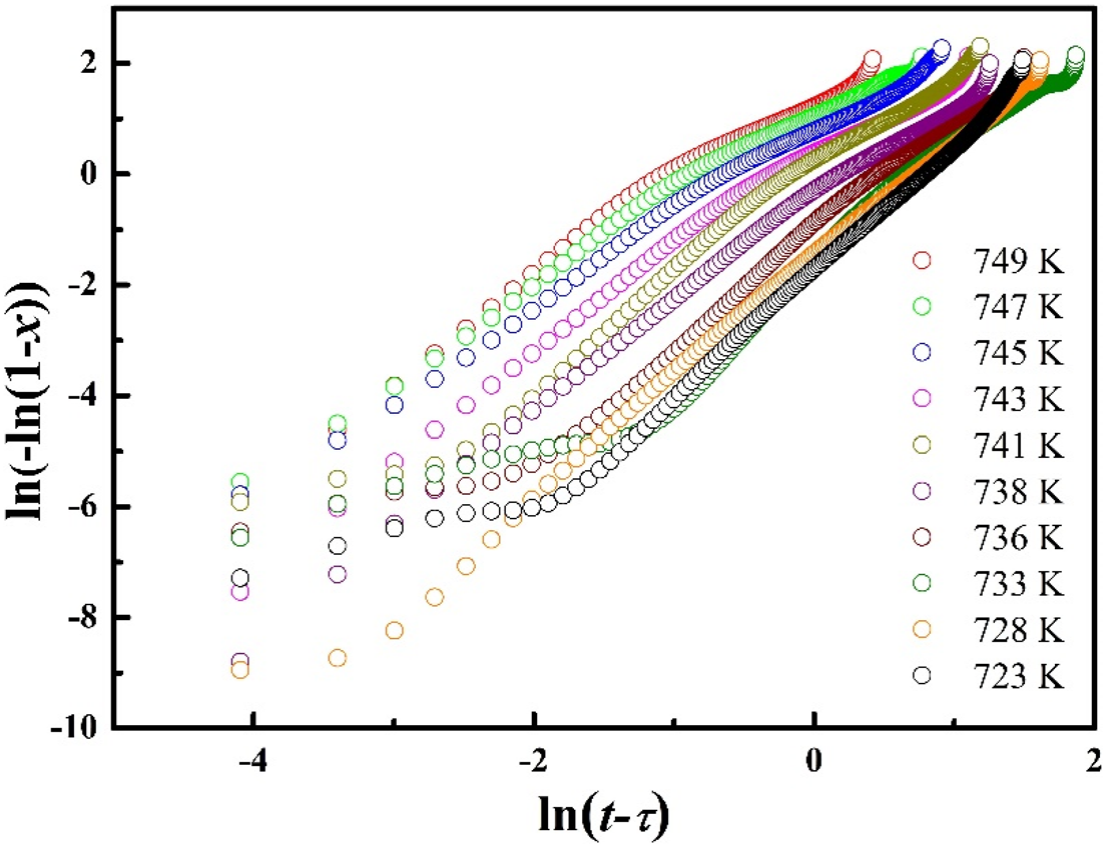
Avrami plots of (Cu_60_Zr_25_Ti_15_)_99.3_Ni_0.7_ BMG at various annealing temperatures.

**Figure 8:**
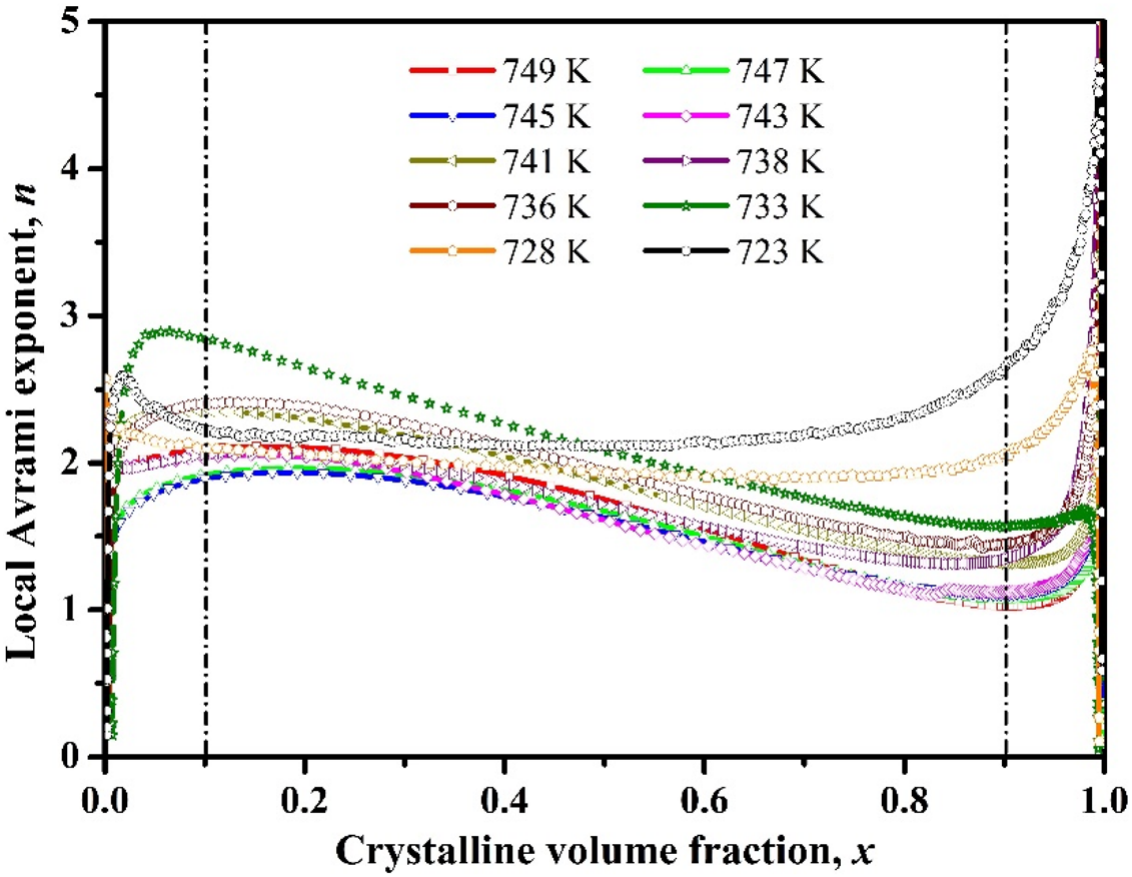
The Avrami exponents with crystallized fraction derived from the JMA equation.

Thus, the kinetic parameters *n* and *K* are obtained by plotting ln(–ln(1–*x*)) against (t–*τ*), as shown in Fig. 7. The trend of the nucleation and growth is impulsive during the crystallization process. The correlation between local Avrami exponent *n* and the crystallized volume fraction *x* are shown in Fig. 8. The Avrami exponent gives more realistic understanding about the crystallization kinetics^54^. The local Avrami exponent *n* at the different phases of the crystallization can be obtained from the following equation^43,54,55^:

4 $$n(x) = \frac{{\partial \ln \{ - \ln (1 - x)\} }}{\partial \ln (t - \tau )}$$

It is well known that, the local Avrami exponent *n* is interrelated to the mechanisms of nucleation and growth behavior of BMGs. Moreover, the slope of the Avrami plots *n* can be determined by the following equation^54,55^:

5 $$n = b + pm$$

where, *b* is the nucleation index (constant nucleation rate *b* = 1; zero nucleation rate *b* = 0; *b* > 1 indicates about the growing nucleation rate, and 0 < *b* < 1 indicates about the decreasing nucleation rate with time^55^. The 1-, 2- and 3- dimensional grain growth are indicated by *m* with the values of 1, 2, and 3, respectively^54^. The *p* symbolizes the growth index where, *p* = 1 indicates about the interface controlled growth and *p* = 0.5 points to the diffusion controlled growth^54^.

Figure 8 reveals that, irrespective of temperature, the Avrami exponent varies with the crystallized volume fraction. The Avrami exponent *n* value is in between 1.5 and 2.5 at the beginning of the crystallization (*x* = 0). This indicates about the diffusion controlled mechanism of the nucleation from the liquid phase. If we consider the crystallization pathway in between the 10% and 90% crystallized volume fraction, the Avrami exponent continuously decreases upto ~ 1.5 at the final stage of crystallization. This demonstrates that the nucleation rate is reducing almost upto zero. At the final stage of the crystallization process (*x* > 90%), Avrami exponent is increased, which corresponds to the increasing rate of nucleation in the diffusion-controlled three-dimensional growth. The *n*_*average*_ values are relatively similar for all of the annealing temperatures and indicates about the three dimensional growth with decreasing nucleation rate. It is reported that crystallization mechanisms are dependent on the temperature and vary from interface controlled to diffusion-controlled crystallization mechanism at different annealing temperatures^56^.

### Continuous heating transformation

**Figure 9:**
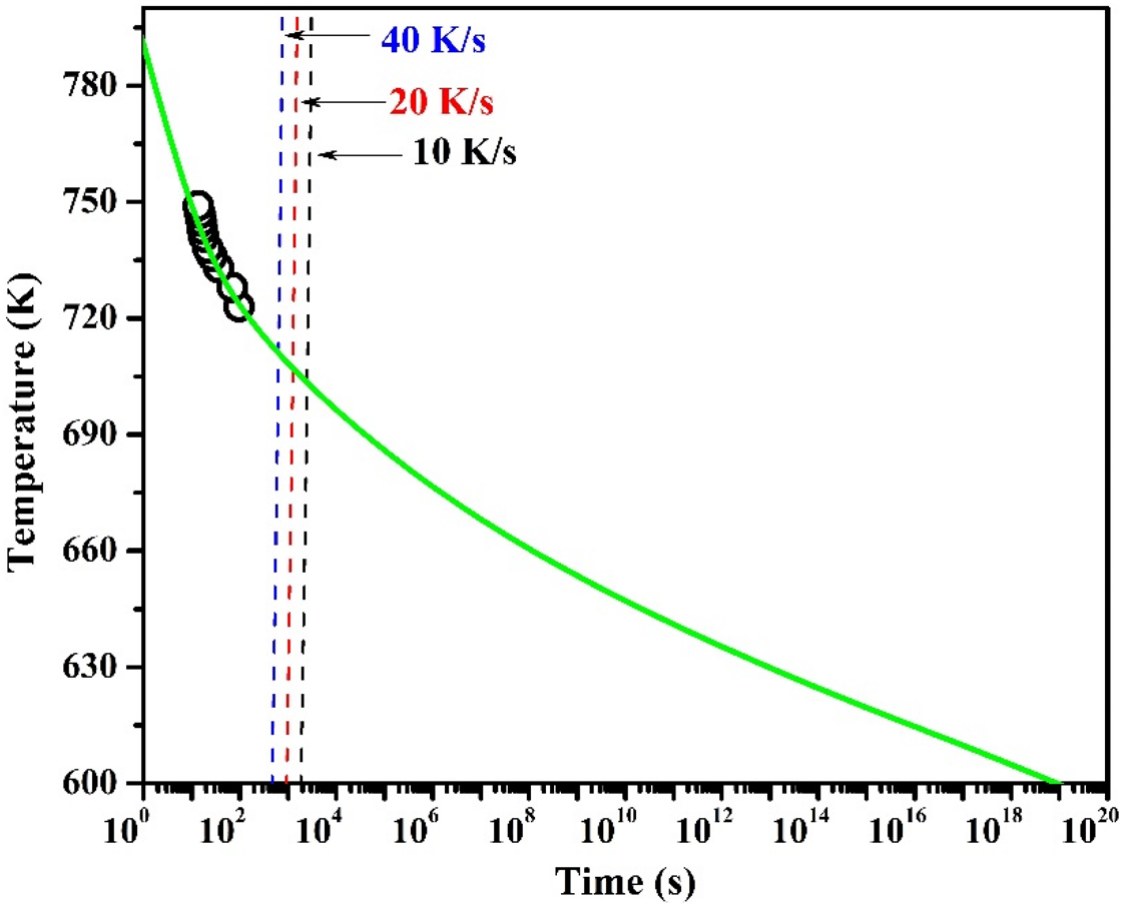
Continuous heating transformation (CHT) diagram for primary crystallization of (Cu_60_Zr_25_Ti_15_)_99.3_Nb_0.7_ bulk metallic glass. The vertical lines indicate the heating curves at 10–40 K/min heating rates.

To understand the thermal stability, the time dependent phase transformation of (Cu_60_Zr_25_Ti_15_)_99.3_Nb_0.7_ bulk metallic glass has been presented as a continuous heating transformation (CHT) diagram. Similar studies on the CHT of MGs/BMGs have reported earlier by Yang et al. (2016) and Louzguine et al. (2002) for Zr-based and Cu-based systems respectively^57,58^. The continuous heating transformation diagram has been made by plotting the transformation start temperature against the time required for the transformation to start for the isothermal heat treatments of the BMG sample. The Fig. 9 shows the CHT plot where, the time axis is in logarithmic coordinate and Y-axis shows the temperature. The green-colored solid curve represents the *T*_*o*_-*t*_*h*_ relationship, while the black, red and blue dashed curves depict the heating program in DSC experiments with 10, 20, and 40 K/min heating reates respectively. The dashed lines are made by calculating the time required for the mentioned heating rates and the intersections of the dashed lines with the solid green line indicates about the time required to reach that particular temperatre. With the increasing set temperature for isothermal annealing, the time required for the transformation start is less. The heating rate (*β*) to reach any transformation temperature may be expressed as^57,58^:

6 $$\beta = T_{o}^{2} e^{{b/T_{o} + c}}$$

where, *T*_*o*_ is the onset of crystallization peak temperature, *b* and *c* are the gradient and intercept, respectively.

The heating time (*t*_*h*_) required to reach *T*_*o*_ from room temperature (i.e., 298 K) can be obtained from^57,58^

7 $$t_{h} = (T_{o} - 298)/\beta$$

From the extrapolation of the solid green curve, it is very clear that (Cu_60_Zr_25_Ti_15_)_99.3_Nb_0.7_ bulk metallic glass remains stable and unchanged at room temperature and to reach the *T*_*o*_ at 600 K (much lower temperature than the *T*_*o*_), it will take more than 10^11^ years.

Applying the similar method, Louzguine and Inoue^58^ reported a temperature decreases up to ~ 600 K in over 10^5^ years for Cu_60_Hf_25_Ti_15_ MG^58^. Yang et al. (2016) reported about the room temperature stability for over 10^20^ years of ZrTiCuNiBe, ZrTiHfCuNiBe high entropy BMGs, and 10^10^ years for Zr_41.2_Ti_13.8_Cu_12.5_Ni_10_Be_22.5_ BMG^57^.

### Identification of precipitated phases

**Figure 10:**
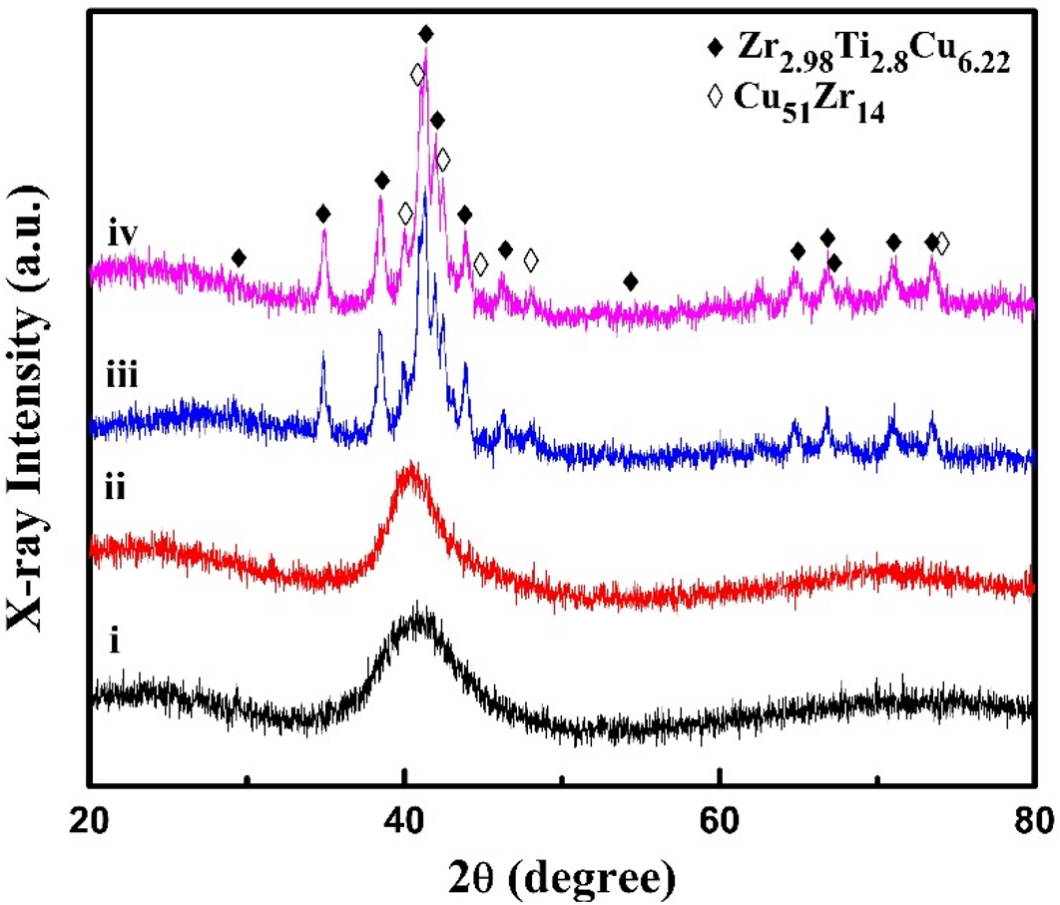
XRD patterns of (Cu_60_Zr_25_Ti_15_)_99.3_Nb_0.7_ alloys as cast (i); heat treated up to peak temperature (ii); 10 min hold at peak temperature (iii); 10 min hold at peak completion temperature (iv).

In order to examine the crystalline phases evolved during the isothermal annealing process, X-ray diffraction study of the (Cu_60_Zr_25_Ti_15_)_99.3_Nb_0.7_ glass sample has been done. XRD patterns of as cast and heat treated (Cu_60_Zr_25_Ti_15_)_99.3_Nb_0.7_ BMG at different temperatures are presented in Fig. 10.

Temperatures for heat treatments have been selected at the peak temperature, 10 min hold at peak temperature and 10 min hold at peak completion temperature and the plots are labelled as ii, iii, and iv, respectively. The XRD scan labelled as i, is the scan of the as cast (Cu_60_Zr_25_Ti_15_)_99.3_Nb_0.7_ BMG re-presented here for the comparison. In spite of heating the sample till the temperature corresponding to peak, the XRD pattern has only an amorphous hump (with no sharp peaks) similar to that of the as-cast sample; however, an increase in the intensity and a decrease in the width of the amorphous hump with respect to the as-cast sample are observed. This may be due to the formation of very few and small nanocrystals inside the amorphous matrix which are beyond the detectable limit of the XRD^59–61^. This also depicts the thermal stability of the (Cu_60_Zr_25_Ti_15_)_99.3_Nb_0.7_ BMG. However, isothermal holding at this temperature for ten minutes has caused the crystallization to commence as indicated by the small diffraction peaks emerging from the amorphous background. Majority of the small diffraction peaks correspond to Zr_2.98_Ti_2.8_Cu_6.22_ phase (PDF 01-076-7199) and the remaining correspond to Cu_51_Zr_14_ (PDF 04-003-0313) phase. Diffraction peaks for the sample heat treated till the completion of the exothermic peak (even after heating for 10 min at this temperature) has appeared at the same positions as that of the sample heat treated for ten min at the peak temperature; this means no new phase other than Cu_51_Zr_14_ and Zr_2.98_Ti_2.8_Cu_6.22_ is formed at the completion of the crystallization.

## Conclusion:

The isothermal crystallization kinetics of (Cu_60_Zr_25_Ti_15_)_99.3_Nb_0.7_ BMG has been investigated using DSC. The conclusions of this study are as follows:

- The sigmoidal type curves of crystallized volume fraction against time become steeper with the higher annealing temperatures, and it demonstrates about the sharp crystallization with shorter time at the higher annealing temperatures.
- Continuous heating transformation (CHT) diagram has been constructed and the long stability at the room temperature has been predicted for the (Cu_60_Zr_25_Ti_15_)_99.3_Nb_0.7_ BMG from the CHT plot.
- The activation energy (*E*_a_) calculated by the Arrhenius equation shows that the *E*_a_ values decreases constantly from 362 kJ/mol to 303 kJ/mol. So, the activation energy required for nucleation, *E*_*nucleation*_ is greater than the activation energy required for the growth, *E*_*growth*_.
- The Avrami exponent values, *n* indicates about the diffusion controlled mechanism of the nucleation from the liquid phase at the beginning and at the end, an increasing nucleation rate in the diffusion-controlled three-dimensional growth.
- The XRD result indicates that Zr_2.98_Ti_2.8_Cu_6.22_ and Cu_51_Zr_14_ phases have been precipitated during the isothermal annealing of (Cu_60_Zr_25_Ti_15_)_99.3_Nb_0.7_ BMG and shows very good thermal stability against crystallization as there is no peak found after the heat treatment upto the peak temperature.
